# The impact of income definitions on mortality inequalities

**DOI:** 10.1016/j.ssmph.2021.100915

**Published:** 2021-09-07

**Authors:** Jiaxin Shi, Lasse Tarkiainen, Pekka Martikainen, Alyson van Raalte

**Affiliations:** a Max Planck Institute for Demographic Research, Rostock, Germany; b Leverhulme Centre for Demographic Science, Department of Sociology, University of Oxford, Oxford, United Kingdom; c Population Research Unit, University of Helsinki, Helsinki, Finland; d Department of Public Health Sciences, Stockholm University, Stockholm, Sweden

**Keywords:** Health and mortality inequality, Measurement, Data quality, Life expectancy, Administrative data, Finland

## Abstract

Income is a strong predictor of adult mortality. Measuring income is not as simple as it may sound. It can be conceptualized at the individual or the household level, with the former better reflecting an individual's earning ability, and the latter better capturing living standards. Furthermore, respondents are often grouped into income categories based on their positions in the income distribution, and this operationalization can be done on the basis of age-specific or total population income distributions. In this study, we look at how four combinations of different conceptualizations (individual vs. household) and operationalizations (age-specific vs. total population) of income can affect mortality inequality estimates. Using Finnish registry data, we constructed period life tables for ages 25+ from 1996 to 2017 by gender and for four income definitions. The results indicated that the slope index of inequality for life expectancy varied by 1.1–5.7 years between income definitions, with larger differences observed for women than for men. The overall age patterns of relative index of inequality for mortality rates yielded by the four definitions were similar, but the levels differed. The period trends across income definitions were consistent for men, but not for women. We conclude that researchers should pay particular attention to the choice of the income definitions when analyzing the association between income and mortality, and when comparing the magnitude of inequality across studies and over time.

## Highlights

• We examined how income definitions impact estimates of mortality inequality by income.
• Different income definitions substantially affected the level of mortality inequality.
• Period patterns across income definitions were consistent for men but not for women.
• Age patterns of mortality inequality were consistent across income definitions.
• The overall impact of income definitions is larger for women than for men.

## 1 Introduction

Researchers have long been interested in the association between socioeconomic status and adult morbidity and mortality (Karisto et al., 1978; Kitagawa & Hauser, 1973; Stockwell, 1963). In particular, with high-quality administrative data, a growing body of research investigates the association between income and mortality risks or life expectancy (e.g., Brønnum-Hansen, H., & Baadsgaard 2012; Tarkiainen et al., 2012; Tarkiainen et al., 2013; Chetty et al., 2016). While these studies have measured income in different ways, how the definition of income affects the estimation of health inequality is less understood. It is often of scholarly interest to compare health and mortality inequality levels across countries or over time by comparing results from different studies. The inequality patterns including levels and trends depend on the exact income definition used by researchers. The various choices of income definitions challenge the comparability across studies.

The aim of this paper is to improve our understanding of how the definition of income researchers use affects the income-mortality associations they observe. To do so, we make use of novel administrative data on the entire Finnish population spanning more than two decades. We focus on four commonly used income definitions that concern two conceptualizations (individual vs. household) and two operationalizations (population-wide vs. age-specific income cut-points), and examine how the use of different income definitions leads to different estimates of mortality inequality over time.

## 2 Background

### 2.1 Common income definitions

The four income definitions we examine pertain to the conceptualization and operationalization of income. First, income can be conceptualized at the individual or the household level. Focusing on individual income, either gross or net (disposable), is one way of conceptualizing income in analyses of mortality inequality (Waldron, 2007; Wilkins et al., 1989). Alternatively, household income, which combines the income of all the individuals living in the same household, may be used in the analyses. Some previous studies have used total household income, either pre- or post-tax (Chetty et al., 2016; Dowd et al., 2011; Hederos et al., 2018; Luy et al., 2015). Household disposable income per consumption unit (hereafter, household disposable income) is a definition that is often employed in these studies. This definition accounts for all household members' income, as well as the economies of scale that come from adding members to the household (Brønnum-Hansen, H., & Baadsgaard 2012; Henriksson et al., 2006; Kinge et al., 2019; Ng et al., 2020; Östergren et al., 2019; Shi et al., 2021).^1^

Second, when individuals are divided into specific income percentiles—an approach that is often adopted to divide individuals into equally sized ordinal groups, instead of categorizing them into absolute income brackets—there are two main ways that income is operationalized. One is to assign individuals to percentile groups based on their positions in the age-specific (e.g., one-year age group) income distributions; i.e., to use age-specific cut-points (Kinge et al., 2019; Shi et al., 2021). This way, the different income groups have the same number of observations for each age group. The other approach is to divide individuals on the basis of income cut-points derived from the income distribution of the total population, while ignoring age differences in income (Hederos et al., 2018; Luy et al., 2015). By doing so, young adults and retirees tend to be in lower-income groups, as income generally increases with age, peaks at middle age, and declines thereafter (Hedström & Ringen, 1987; Lee & Mason, 2007). We therefore posit that using population cut-points at the two ends of the adult age range, lower-income groups will tend to have more deaths and exposures than higher-income groups.

Admittedly, the challenges of measuring income are not just related to the aforementioned choices. A broad literature on income inequality has looked at other dimensions of income definition as well. For example, the composition of total income (e.g., capital versus labor income) varies across the income distribution (Ranaldi, 2021), and different income components may affect health differently. Another conceptual consideration is that individuals differ not only in their annual income, but also in their long-term income or the characteristics of their income trajectory (Frech & Damaske, 2019). In terms of the grouping strategy, individuals can be assigned to income groups based on their percentiles in gender-specific or gender-combined income distributions. In this paper, we do not compare different income components or trajectories, but instead focus on the definitions that are commonly found in the mortality inequality literature, while using gender-specific income distributions.

### 2.2 Theoretical considerations

The absolute income theory posits that individuals’ incomes affect their health and mortality because of its absolute value (Preston, 1975; Rodgers, 1979). By contrast, the relative income theory suggests that it is not the actual amount of money individuals have that determines how much mortality risk they face, but rather their relative positions on the social ladder (Kawachi et al., 2010; Wagstaff & Van Doorslaer, 2000). Essentially, the income definitions that we consider here are all on the relative scale due to groupings based on percentiles, but some capture the absolute aspect of income more than others.

Household disposable income better captures actual living standards than individual income, because incomes are usually pooled within the household. The use of individual income can be especially problematic for capturing material conditions of partnered individuals who do not work. For example, a woman outside of the paid workforce with a high-income husband may have a very low individual income, but adequate economic resources. However, the use of individual income also has advantages, as over working ages, it arguably captures the earning ability of most men in the labor market better than household income. However, because of strongly gendered child care this is not necessarily the case for many women.

If the measurement of income is intended to capture earning ability, the use of individual income might be more suitable when studying men than when studying women, as a larger share of working-age women have part-time jobs. This is particularly the case for trend studies conducted in settings where female labor force participation behavior has changed. If income is intended to capture material circumstances, consumption ability, and living standards, household disposable income may be preferred over individual income, as it takes into account the within-family joint use of economic resources.

Population-wide grouping might be preferred as an indicator of actual material circumstances and absolute income. It does not consider changes in income over the life course. Although the income some individuals need to maintain their standard of living declines somewhat at older ages because prior savings (e.g., private saving for retirement), lower housing costs (e.g., among homeowners, mortgages tend to be paid off), and work-related costs are lower, the replacement rate needed to achieve equivalent living standards is higher among low-income earners, who typically save less and rent more (Scholz & Seshadri, 2009). Thus, the income percentile cut-offs that reflect the ability of individuals to maintain a healthy standard of living might be more appropriately assessed at the population level, since they are more likely to be influenced by overall macro-level factors than by age alone. On the other hand, age-specific grouping better captures the relative aspect of income. As people tend to compare themselves to others with similar social experiences (Kawachi & Subramanian, 2014), relative income positions are arguably better captured by comparing individuals with those of similar ages.

How researchers group income will define the aspects of inequality they will capture. Different definitions of income reflect the theoretical links between income and mortality to varying degrees. Researchers should consider the possible implications of these differences, and select a definition of income based on theoretical considerations.

### 2.3 Objectives

Our main objective is to provide empirical evidence on how the definition of income used in analyses of mortality inequality affects the robustness of the income-mortality associations. This is an important issue that has received little attention in the existing literature. One previous study using regression models showed that individual income is more closely associated with survival than household disposable income at the individual level (Martikainen et al., 2009). While prior aggregate-level research has discussed the potential impact of a specific income conceptualization (e.g., Hederos et al., 2018; Ng et al., 2020), to date, no aggregate-level study has extensively examined how the income definition chosen affects the inequality results. To address this question, we put forward the following hypotheses.

*Magnitude and rankings.* As different income definitions assign individuals to different population subgroups, we expect to find that group-specific mortality and longevity outcomes differ in magnitude depending on the income definition used. Thus, the use of different definitions may lead to different mortality rankings, and, in turn, different mortality inequality levels.

*Age and period patterns*. Age and period trends may vary between different definitions of income. The age and period patterns we examine address two related issues. One is whether different income definitions yield the same age patterns and period trends (i.e., increasing, decreasing, or leveling-off). The other issue is more nuanced: namely, does the ranking of mortality inequality by income definition vary across ages and periods? Essentially, we expect to observe that when different income definitions are used, mortality inequalities of varying magnitudes are found. Moreover, we examine whether these differences are age- and period-dependent.

*Gender differences*. Socio-economic differences in total mortality are usually larger for men than they are for women. While this is likely to be true regardless of the income definition that is used, other gender differences may emerge. We have outlined several potential gender differences in the previous subsection. The most critical difference appears to arise depending on whether a definition based on household or individual income is used. Due to gendered division of labor force participation, gender differences in pay and labor within households, a partnered woman with low individual income is more likely to enjoy relatively good material circumstances as compared to a non-partnered woman. Hence, the use of household income may have more discriminatory power than the use of individual income to differentiate women's standard of living, and thus may lead to larger mortality inequalities. This is not necessarily the case for men. In addition, gender differences may also interact with age and period dimensions. In older ages, when gender differences in income become smaller, the impact of income definitions on mortality inequalities may also be smaller.

## 3 Material and methods

Our analysis uses Finnish administrative data. Specifically, income information for all Finns aged 25 and above covering the period of 1995–2017 was provided by the Finnish Tax Administration and the National Social Insurance Institution. Moreover, death records for the same period were obtained from the death registry. The information from these two sources was then linked by Statistics Finland using a unique personal ID, which is assigned to all permanent residents of Finland, and is used in all registries. An additional household ID was used to identify individuals who live in the same household. In the next step, we divided death counts and exposures (i.e., person-years) for each year based on the income from the previous year. Thus, we have death counts and exposures for each gender, income (corresponding to the specific income definition), year (between 1996 and 2017), and one-year age group (from ages 25–26 to the last open-ended age group of 100+). To tackle the problems caused by data sparseness at certain ages, we used a P-spline model to smooth death counts with exposures as offsets, and to obtain smoothed age-specific mortality rates (Eilers & Marx, 1996). The smoothing method was implemented using the “MortalitySmooth” *R* package (Camarda, 2012).

We examined the four definitions of income that are most commonly used in the health inequality literature: individual income with age-specific grouping, individual income with population-wide grouping, household disposable income with age-specific grouping, and household disposable income with population-wide grouping (in the population ages 25+). For 0.7% of the person-years, no information on individual income was found in the registers, and these person-years were excluded from the individual income analysis. Information on household income was available for all individuals residing in private households. For the non-household population (e.g., institutionalized persons), individual income was used as their household income. Individual and household income refers to net income, i.e., income after tax and including taxable and non-taxable income transfers. Household disposable income was calculated using the OECD-modified equivalence scale (OECD, 2013). Specifically, we divided the total net household income by the sum of consumption units. We assigned one to the first adult, 0.5 to each additional adult member, and 0.3 to each child (Hagenaars et al., 1994). In line with previous studies, we divided individuals into five quintile groups (Auerbach et al., 2017; Brønnum-Hansen, Östergren, et al., 2021; Hederos et al., 2018; Tarkiainen et al., 2013). Then, life tables were calculated for each period, gender, and income quintile based on the four definitions.

Population health researchers have long been using two types of measures to compare mortality across social groups: namely, mortality rates and life expectancy (Antonovsky, 1967; Kitagawa & Hauser, 1973). Researchers often calculate mortality rate ratios to indicate the relative differences between two groups. Alternatively, mortality rate differences show the actual sizes of the differences. Likewise, life expectancy can be compared through either ratios or differences, while the latter is often preferred in the literature (e.g., Brønnum-Hansen, Östergren, et al., 2021; Shi et al., 2021).

We used the regression-based measures slope index of inequality (*SII*) and the relative index of inequality (*RII*) to bring together results for all five income groups, and to assess the inequality levels (Regidor, 2004). First, we assigned a value to each income group that represents the cumulative mid-point income position of that group. Hence, 0.1, 0.3, 0.5, 0.7, and 0.9 were assigned to groups from the lowest quintile to the highest quintile, respectively. Second, we fitted linear models using the ordinary least squares method with the mortality indicator (life expectancy or log age-specific mortality rate) as the dependent variable, and the mid-point income position value as the independent variable. We then predicted the outcome variable for the independent variable taking the value of zero and one, denoted as *Y*_*X = 0*_ and *Y*_*X = 1*_. For life expectancy, the *SII* was calculated as *Y*_*X = 1*_– *Y*_*X = 0*_, and the *RII* was calculated as *Y*_*X = 0*_/*Y*_*X = 1*_; for the log age-specific mortality rate, the *SII* was calculated as *Y*_*X = 0*_ – *Y*_*X = 1*_, and the *RII* was calculated as *Y*_*X = 1*_/*Y*_*X = 0*_.

Analogous to the rate (or life expectancy) difference, the *SII* is an absolute measure, whereas the *RII* is a relative measure that resembles rate (or life expectancy) ratios. The *SII* and the *RII* can be interpreted as the absolute and the relative difference in life expectancy or mortality rates between individuals with the lowest to the highest positions on the income ladder. The *SII* normally takes positive values, and a larger *SII* means greater inequality in absolute terms; *RII* normally takes values over 1 and larger *RII* means larger inequality in relative terms. However, when the association between income and mortality outcomes is negative, the *SII* is negative and the *RII* is between zero and one. In the following section, we show the *SII* for life expectancy and the *RII* for mortality rates, as researchers are often interested in the absolute difference in life expectancy and the relative difference in mortality rates. Overall, the *SII* and the *RII* show very similar age and period patterns for both mortality outcomes; the complete results are presented in the appendix.

## 4 Results

### 4.1 Compositional differences

**Fig. 1:**
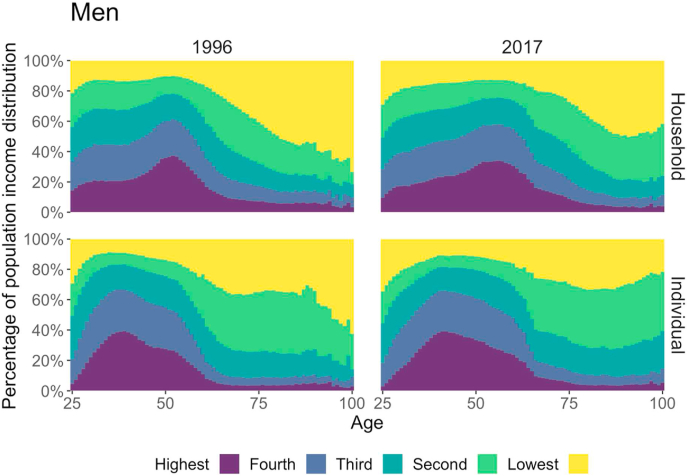
**Age-specific percentage of individuals by income group based on gender-specific population income distribution, men, 1996 (left column) and 2017 (right column).** The upper row panels adjust for household size (i.e., household disposable income), and the lower row panels show individual income. *Source:* Authors' calculation based on Finnish registry data.

Fig. 1 (men) and 2 (women) show the percentages of individuals who fell into different quintile groups at a given age, and how these percentages changed with age when using the population-wide grouping for 1996 and 2017, and for two conceptualizations. For the age-specific grouping, different income groups occupied the same fractions (20%) at all ages, and we would observe parallel boundaries at the 20%, 40%, 60%, and 80% positions in these graphs.

**Fig. 2:**
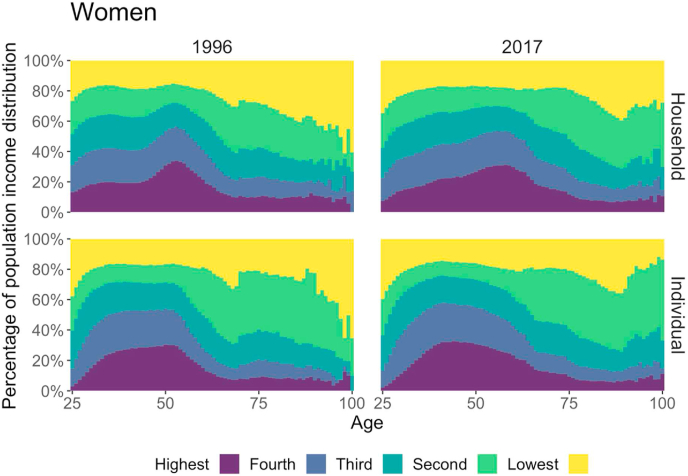
**Age-specific percentage of individuals by income group based on gender-specific population income distribution, women, 1996 (left column) and 2017 (right column).** The upper row panels adjust for household size (i.e., household disposable income), and the lower row panels show individual income. *Source:* Authors' calculation based on Finnish registry data.

Fig. 1, Fig. 2 show that the boundary lines are far from the parallel lines in the age-specific grouping. Put differently, we can see large differences in individual compositions for the two operationalizations, which suggests that the two operationalizations may rank individual mortality outcomes differently. Individuals of prime working ages (around 50) were more likely to be in the highest income quintile than individuals in any other age group. As individuals grew older and started entering retirement, they became increasingly likely to be in the bottom quintile, and only a small number of them remained in the highest quintile. This was particularly the case for men in 1996 when using household income. Similarly, younger adults tended to earn less than middle-aged people, and individuals at the lower end of our age range were unlikely to be in the top income quintile. This was particularly the case for women when using individual income.

**Fig. A1:**
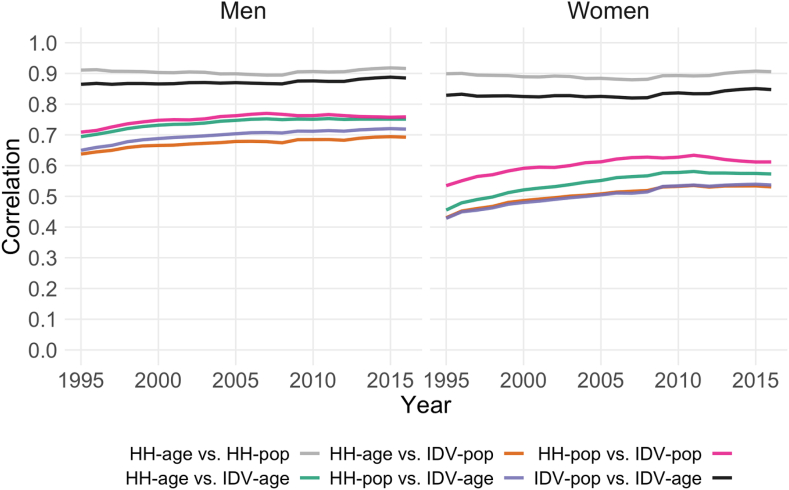
**Correlation coefficients between income definitions 1996**–**2017.***Source:* Authors' calculation based on Finnish registry data, 1995–2017. *Note:* Abbreviations are explained in brackets as follows: HH (household disposable income), IDV (individual income), age (age-specific income distribution), pop (population income distribution).

Fig. 1, Fig. 2 indicate that individuals were re-allocated from one quintile to another when a different income definition was used. To explore how frequently this happened and how similar the definitions were, we calculated the correlation coefficients of individuals’ quintile groups for each year and for each of the two income definitions (Table 1). The correlation coefficients were found to be relatively stable across the years. The year-specific correlation coefficients presented in Figure A1 show that the conceptualization mattered more than the operationalization. When only the operationalization differed, the correlations were high; when only the conceptualization differed, the correlations were medium; and when both differed, the correlations were low (Fig. A1). These results confirm that individuals fell into different income quintiles depending on the income definition.

**Table 1:** Correlation coefficient between income definitions by gender.

Variables	Household, age-specific	Household, population-wide	Individual, age-specific	Individual, population-wide
*Men*				
Household, age-specific	1.00			
Household, population-wide	0.91	1.00		
Individual, age-specific	0.74	0.70	1.00	
Individual, population-wide	0.68	0.75	0.87	1.00
*Women*				
Household, age-specific	1.00			
Household, population-wide	0.89	1.00		
Individual, age-specific	0.55	0.50	1.00	
Individual, population-wide	0.51	0.60	0.83	1.00

### 4.2 Differences in life expectancy levels and trends

**Fig. 3:**
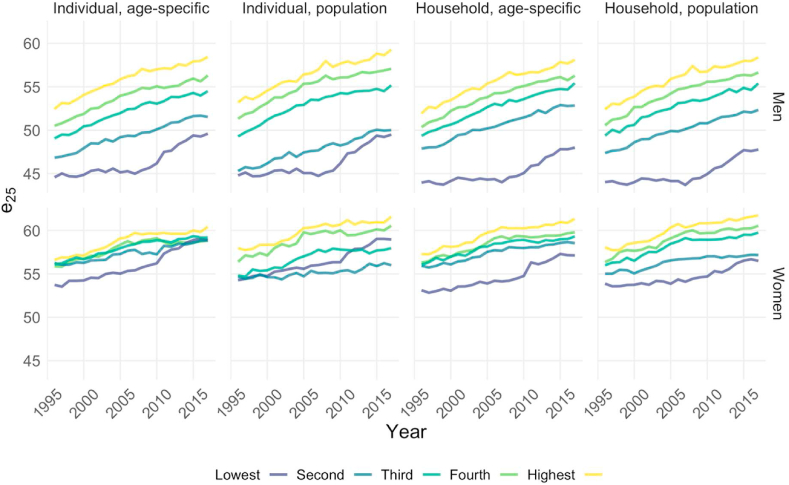
**Life expectancy at age 25 by income quintile, 1996**–**2017.***Source:* Authors' calculation based on Finnish registry data, 1995–2017.

Fig. 3 shows trends in life expectancy at age 25 by gender for each income definition. For all of the definitions, substantial differences in life expectancy at age 25 by income were found for all years. A positive association between income and life expectancy was found in almost all panels, with one exception for women: i.e., when individual income was used, the lowest income quintile caught up with the higher income quintiles in more recent years, especially for the population-wide grouping. On the other hand, for women, the use of the age-specific grouping for individual income was associated with much less variation in life expectancy than the use of the population-wide grouping. While we did not observe these patterns among men, we did find that the gap between the lowest-income men and higher-income men became smaller when individual income was used, especially for the population-wide grouping.

**Fig. 4:**
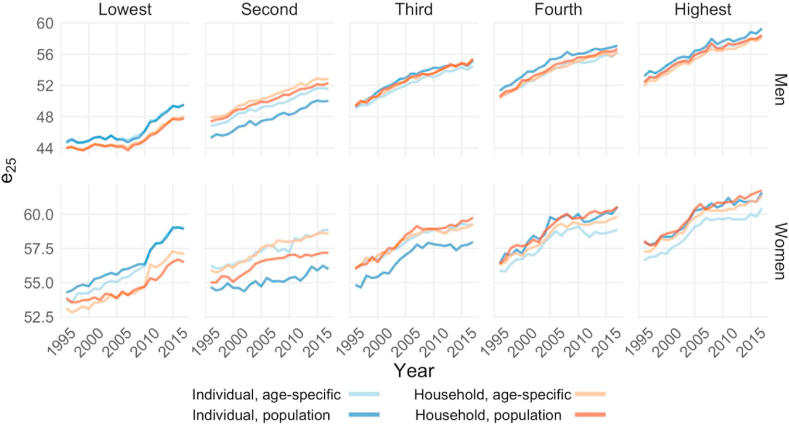
**Life expectancy at 25 by income definition, 1996**–**2017.***Source:* Authors' calculation based on Finnish registry data, 1995–2017. *Note:* The scales for the first and the second row are different.

Fig. 4 shows how the use of different income definitions affected the life expectancy levels and trends found for each income group. For the bottom income group and for both men and women, higher life expectancy levels were found when individual income was used than when household disposable income was used. For the higher-income groups, higher life expectancy levels were generally found when household disposable income was used than when individual income was used, although this pattern was reversed for the highest two male income groups. The difference in estimated life expectancy when using one definition of income rather than another was non-negligible, as it was as large as 2.5 years for certain quintiles in certain years. In short, the use of different income definitions led to substantial differences in the levels of life expectancy for both genders, and it altered the quintile-rankings of life expectancy for women. Fig. 4 also shows that the income definition used mattered more for women than for men.

### 4.3 Differences in inequality estimates

**Fig. 5:**
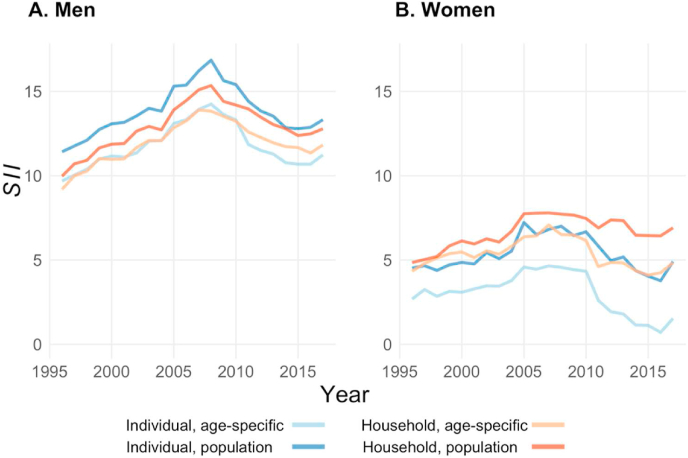
**Trends in the slope index of inequality (*SII*) for life expectancy by income definition and gender, 1996**–**2017.***Source:* Authors' calculation based on Finnish registry data.

**Fig. A2:**
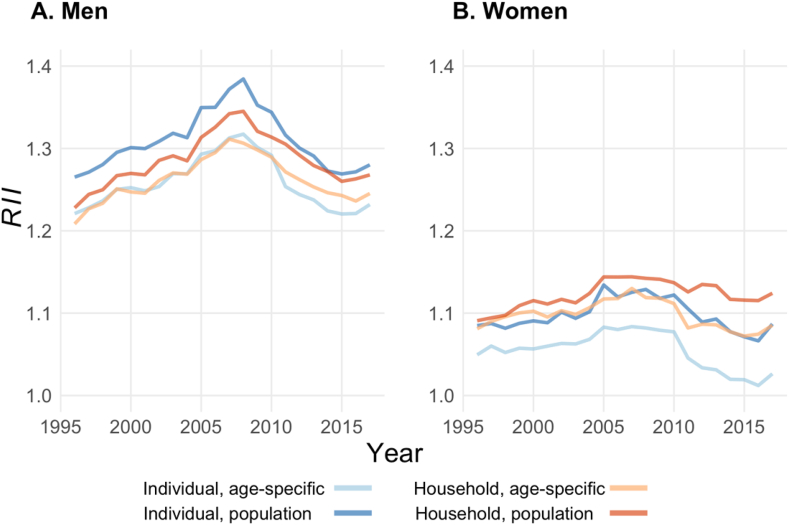
**Trends in the relative index of inequality for life expectancy by income measure and gender, 1996**–**2017.***Source:* Authors' calculation based on Finnish registry data, 1995–2017.

Fig. 5 shows the *SII* for life expectancy (*RII* in Fig. A2).^2^ In 1996, the *SII* was less than five years for women (i.e., life expectancy of women at the top of the income distribution was almost five years higher than the life expectancy of those at the bottom), and was around 10 years for men. For men, similar temporal *SII* trends for life expectancy were found for all four income definitions, with inequality increasing between 1996 and 2008, and decreasing after 2008. On the other hand, rather different trends were found for women, as household income was shown to stagnate in recent years for the population-wide grouping, while clear decreasing trends were found for the other three definitions. Fig. 5 also suggests that which conceptualization and operationalization were applied mattered more for women than for men, as we found greater variation in the *SII* across the four income definitions for women.

Over the years, the differences in the inequality levels observed when different income definitions were used ranged from 1.8 to 5.7 for women and from 1.1 to 3.0 for men. For women, the highest inequality levels were detected when household income with population-wide grouping was used. When the same grouping method was applied, household income showed higher inequality levels than individual income. When the same conceptualization was applied, the population-wide grouping method led to larger *SII* than the age-specific grouping method. The *SII* for the individual-level, age-specific grouping was consistently at least one year lower (≈20% lower) than it was for all other groupings, and widened to up to a three-year difference (≈60% lower) in the past decade. The results for men were different. Among men, the use of individual income with the population-wide grouping method led to the highest levels of inequality in life expectancy. However, when the age-specific grouping method was used, the estimates for men were similar regardless of whether household or individual income was used.

**Fig. 6:**
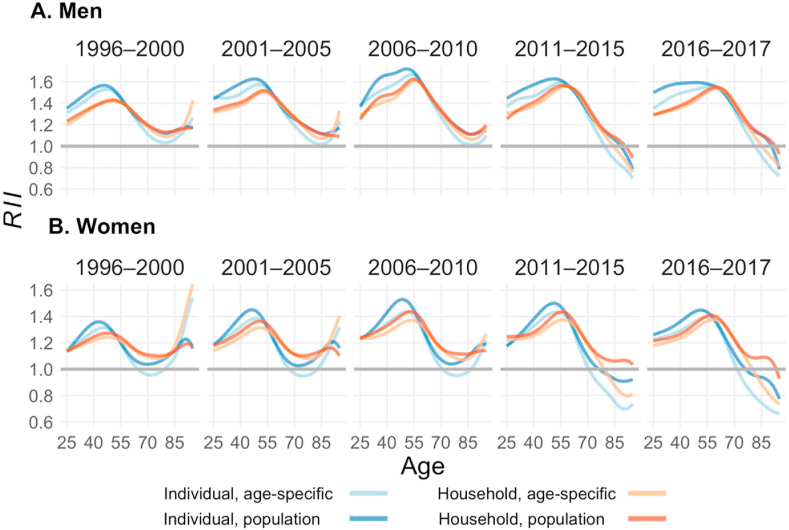
**Relative index of inequality for age-specific mortality rates by period and gender, 1996**–**2017.***Source:* Authors' calculation based on Finnish registry data, 1995–2017. *Note:* The scales for the upper row and the bottom row are different. To illustrate, a level of *RII* of 1.5 here means that the ratio of the log mortality rate between people at the very top and the very bottom of the income distribution is 1.5.

**Fig. A3:**
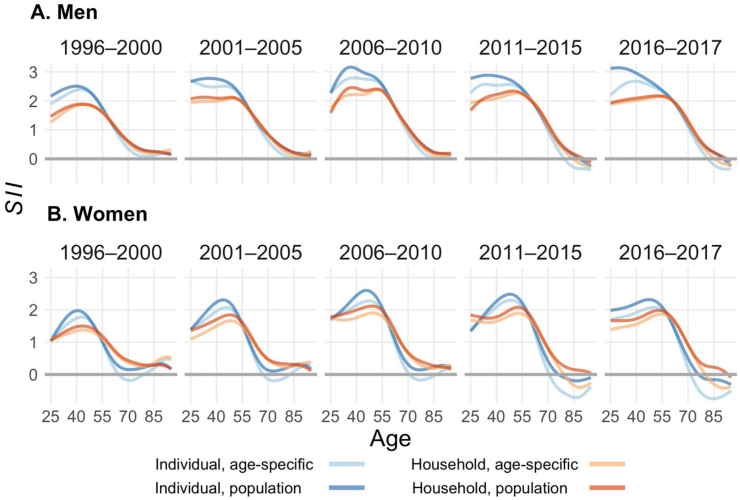
**Slope index of inequality for age-specific mortality rates by period and gender.***Source:* Authors' calculation based on Finnish registry data, 1995–2017. *Note:* To illustrate, a level of *SII* of 2 here means the difference in the log mortality rate between people at the very top and the very bottom of the income distribution is 2.

To better understand how income influenced mortality across age groups, and whether the age patterns of mortality inequality were consistent across income definitions, we present the *RII* for age-specific mortality rates in Fig. 6 (*SII* in Fig. A3). Before the age of 60 for both genders, larger absolute and relative inequalities in mortality rates were found when individual income was used than when household income was used. Beyond this age for women, absolute and relative levels of inequality were lower when individual income was used than when household income was used. While a similar pattern was observed for men, the differences depending on which income definition was used were much smaller for men than for women. The use of household income seemed to attenuate the relationship between income and age-specific mortality. Additionally, inequality levels were shown to be higher for the population-wide grouping than for the age-specific grouping. These patterns were found repeatedly for men and women across all periods.

While the four income definitions were shown to be associated with different levels of inequality, they produced similar age patterns, with inequality first increasing and then decreasing with age. The exact position (age) of the peak depended on the period and the income definition used. The peaks were more likely to occur at older ages when household income was used than when individual income was used. In more recent periods (2011–2015 and 2016–2017), the *SII* became negative at ages above 75. Hence, the association between income and mortality rates was reversed, as higher income was associated with higher mortality rates above these ages. Over time, mortality inequality at ages below 60 first increased and then decreased for both men and women. This pattern was similar to that found for life expectancy differences. However, at older ages, mortality inequalities stagnated in the first three periods and decreased in the last two periods.

## 5 Discussion

Our results indicate that the use of different income conceptualizations and operationalizations can lead to critical differences in estimates of mortality inequality levels. First, we showed that the use of different income definitions led to different estimates of mortality rates and life expectancy levels, and, ultimately, of the levels of inequality between groups. Second, we found that the age patterns in mortality inequalities were consistent regardless of the income definition used. Third, we observed that the period trends of life expectancy differences were similar for men but different for women in more recent years. Fourth, we showed that income groups were ranked consistently across income definitions for men but not for women. A concise overview of the theoretical evaluations and empirical findings for the four income definitions is provided in Table 2.

**Table 2:** Theoretical evaluations and empirical patterns of the four income definitions.

Approach	Conceptual evaluation	Empirical differences and similarities	Gender differences
Conceptualizations			
Household (disposable)	(1) Considers economies of scales; (2) Better reflects material conditions; (3) Better captures absolute income; (4) Unpartnered and unemployed individuals fall into lower income groups.	(1) Larger mortality inequalities at working ages are found when individual income is used than when household income is used; (2) Larger mortality inequalities are found at older ages when household income is used than when individual income is used; (3) The age patterns of inequalities are similar for both, but the use of household income leads to later peaks.	(1) For women, larger differences in life expectancy are found when household income is used than when individual income is used; for men, when population-wide grouping is applied, larger differences in life expectancy are found when individual income is used than when household income is used; (2) Individual income ranks the lowest-quintile women higher than some higher-income groups in recent periods (due to differing household composition); (3) The period patterns for men are similar for both, but the patterns are different for women; (4) Differences in inequality levels depending on the income definitions are larger for women and for men.
Individual	(1) Better reflects earning ability and relative social position; (2) Not able to capture absolute income for partnered individuals who do not work		
***Operationalizations***			
Population-wide grouping	(1) Better reflects absolute income (not wealth or resources); (2) Young and old individuals are less likely to be in high-income groups, and individuals at prime working ages are more likely to be in higher-income groups.	(1) Larger mortality inequalities across ages are found when population-wide grouping is used than when age-specific grouping is used; (2) Larger differences in life expectancy are found when population-wide grouping is used than when age-specific grouping is used; (3) The age patterns are similar for both.	(1) For individual income, the gaps in life expectancy are much smaller when age-specific grouping is used than when population-wide grouping is used, and population-wide grouping ranks the lowest-quintile women much higher. These patterns are not found for men; (2) The period patterns are similar for men, but are different for women; (3) Differences in inequality levels depending on the income income definitions are larger for women and for men.
Age-specific grouping	(1) Better reflects relative positions on income and social ladders.		

We found that the life expectancy ranking was not the lowest for the lowest income quintile when individual income was used. Similarly, using labor earnings, a recent Swedish cohort study also found an unexpected higher ranking in life expectancy for the lowest quintile women (Shi & Kolk, 2021). Further work is needed to better understand the crossover in life expectancy between the lowest and second-lowest individual income groups among women.

The substantial differences in estimates of income-quintile-specific life expectancy make it a challenge for researchers to compare findings from different studies that use different income definitions. While researchers tend to be mostly aware about these measurement issues, journalists may be less so. Reports of life expectancy gaps in the media can stimulate heated public debate. Thus, in studies on life expectancy by income, researchers should provide a clear definition of income, and should explain the potential differences in the life expectancy gap when alternative income definitions are used.

There is a silver lining. Researchers who are interested in the age patterns of the association between income and mortality can be reassured that regardless of the income definition used, the general age patterns are similar: that is, mortality inequality first increases with age until prime working ages, and then decreases rapidly thereafter. As the absolute level of mortality increases with age, it might be counterintuitive to observe a decline in absolute inequality (*SII*). Yet declines in absolute mortality inequality between socioeconomic groups at older ages have been consistently documented in earlier research at ages beyond about 65-years (e.g., Elo & Preston, 1996). In addition, the results provided by the two income conceptualizations converged for men. One potential explanation for this observation is that pension income becomes the main component of income for both individual and household income definitions, and is more homogeneously distributed than pre-retirement wage income, and is less dependent on the individual's health status. However, the exact positions of the age peak in the age-mortality curves differed across definitions, and the peaks tended to occur at older ages when household income was used. In some years, particularly for women, inequality reversed at very old ages. In an earlier Finnish study for the 1991–1996 period, similar age patterns were reported but without the reversal (Martikainen et al., 2001). We do not want to overinterpret the differences in these older age groups, given that at older ages, the absolute sizes of the mortality differences between the income groups were very small.

### 5.1 Household versus individual income

Below age 60, mortality inequalities were larger when individual income was used; whereas above age 60, mortality inequalities were larger when household disposable income was used. At these older ages, when the bulk of mortality occurs, the differences in absolute mortality inequalities between household and individual income were minor for men, but large for women. This gender difference led to different results for the overall levels of life expectancy inequality: i.e., when individual income was used instead of household disposable income, levels of inequality were found to be higher for men but lower for women.

Interpreting the reasons for these different inequality levels is not straightforward. They are determined by the age patterns of mortality rate differences, the sensitivity of life expectancy to age-specific mortality differences (Leser, 1955; Vaupel, 1986), and the composition of each income quintile by age (for population-wide groupings). Moreover, the age patterns of mortality rate differences themselves depend on how the mechanisms (i.e., absolute versus relative income theories) linking income and health vary over the life course. Whether we choose to use one income definition rather than another might depend on whether we assume that absolute or relative income differences are driving inequalities; but even here, the choice between income groupings is not clear-cut.

At working ages, inequalities are generally driven by behavioral risk factors (Nandi et al., 2014; Stringhini et al., 2011), with alcohol-related mortality being a particularly important determinant in the Finnish context (Martikainen et al., 2014; Tarkiainen et al., 2012). Some observers have argued that such behavioral causes of death depend less on overall material conditions, which might be better approximated by household income, and more on coping mechanisms related to stress (Pampel et al., 2010), which might be better signaled by individual income. Thus, our results could be taken to indicate that the role of relative income may be more important at working ages.

Meanwhile, people's earnings ability in the labor market, as indicated by their individual income, also partly depends on their physical and mental health status, and their ability to participate in paid employment. Therefore, pre-existing health problems and other personal characteristics predicting ill health may affect individual income more than household income. This may explain the higher levels of inequality at working ages that we observed when using individual income, given the important role of alcohol-related mortality at these ages, and the substantial declines in income prior to alcohol-related death (Elstad et al., 2019; Tarkiainen et al., 2018). After retirement, emerging new health problems are unlikely to affect their pension incomes. Thus, our finding that at working ages, levels of inequality were higher when individual income was used than when household income was used indicate that the health selection mechanisms were more important at working ages.

Compositional differences in quintile groups over age may also explain the reversal in inequalities across age depending on the income definition. One such compositional factor could be partnership status. While unpartnered women (e.g., divorced women or lone mothers) may experience declines in pension income related to their precarious working histories, for partnered women, career breaks might be less important in determining their income quintile over their pension years when household disposable income is used. Thus, even with similar working histories at younger ages, unpartnered women are more likely than partnered women to be in the lowest quintile group and to experience poor material conditions. Because being unpartnered is a strong risk factor for mortality (Hu & Goldman, 1990), inequalities are larger for women at older ages when household income is used than when individual income is used. Similarly, widowhood is another potential mechanism. Widow(er)s tend to be more concentrated in lower quintiles than non-widow(er)s when household income is used, because widow(er)s are not able to take advantage of economies of scale. As the death of a spouse is a risk factor for mortality (Elwert & Christakis, 2008; Martikainen & Valkonen, 1996), inequality levels are likely to be higher when household income is used than when individual income is used. In terms of individual income, widow(er)s should not differ from non-widow(er)s too much, provided they are not receiving widowhood pensions. The widowhood pension rules differ internationally. As in Finland, the widowhood pension depends both on the income level of the deceased spouse and the widower, the death of a spouse may increase the individual incomes of those in the lowest quintile, and move them to the upper quintile, thus making the lowest quintile a more positively selected (i.e., still partnered) group. This mechanism becomes more important at older ages when widowhood prevalence increases, particularly among women, which is consistent with our findings.

**Fig. A4:**
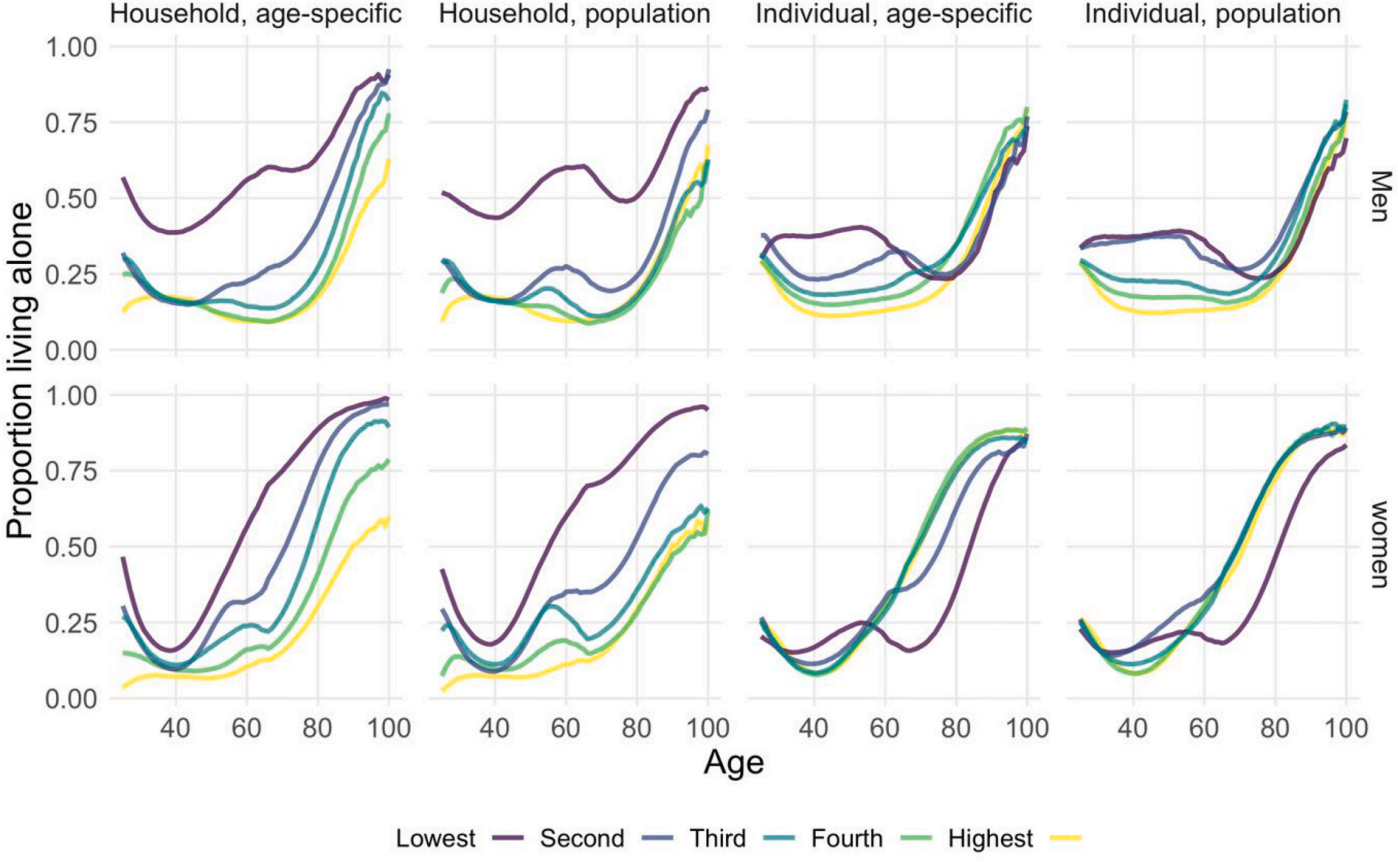
**Proportion of living alone across age (years combined) by income quintile, definition, and gender.***Source:* Authors' calculation based on Finnish registry data, 1995–2017.

In our analysis, the proportion of those who were living alone was highest in the lowest quintile for all income definitions except for individual income among women (Fig. A4). Indeed, when using individual income with population-wide grouping in 2017 for women, we found that at ages above 65, a smaller share of women living alone were in the lowest quintile (≈1/3) than in the other quintiles (≈1/2). It is likely that this discrepancy can partially explain the smaller inequalities for women when using individual income as well as the higher rank of the lowest individual quintile in later periods, as these women bear less often the burden of living alone, and are more likely to be able to take advantage of economies of scale.

Apart from partnership status, employment status might also explain these differences. Being unemployed is a risk factor for mortality (Halliday, 2014; Martikainen, 1990), and unemployed individuals are more likely to end up in lower quintiles if only individual income is taken into consideration. After retirement, not only are individuals out of the workforce, but presumably, even if unemployment carries lingering mortality risks (Garcy & Vågerö, 2012), individuals’ history of periods of employment matters less in determining their income quintiles than their total earned income.

### 5.2 Population versus age-specific cut-points

For both men and women, the differences in mortality rates across all ages were found to be slightly greater for the population-wide grouping than for the age-specific grouping. Small age-specific differences added up to more noticeable differences in life expectancy (1.4–3.4 years using individual income over the period studied). The differences in mortality inequality levels between the two grouping methods were mechanically driven by the differences in the composition of the individuals who fell into the different quintiles. We found that when using the population-wide grouping, the youngest and oldest ages were more likely to be in the lower quintiles than the ages in the middle (Fig. 1, Fig. 2). This pattern can be explained by the well-documented inverted U shape of the income trajectory by age (Hedström & Ringen, 1987; Lee & Mason, 2007). When the age-blind, population-wide grouping approach was applied, individuals in the highest quintile at younger and older ages were positively selected; whereas at the mid-life peak of income earning potential, individuals in the lowest quintile were negatively selected.

Age profiles from national transfer accounts consistently show a comparatively flat consumption pattern over age, particularly as compared to the strong bell-shaped income pattern (Lee et al., 2014). This suggests that population-wide cut-points can better capture material aspects of income, which might not vary strongly by age, while age-specific cut-points are a better marker of relative income, which is highly patterned by age. That inequality levels were higher for the population-wide compared to age-specific grouping method suggests that the material benefits of income might be a more important driver of inequalities than the psycho-social aspects of the relative income perspective.

**Fig. A5:**
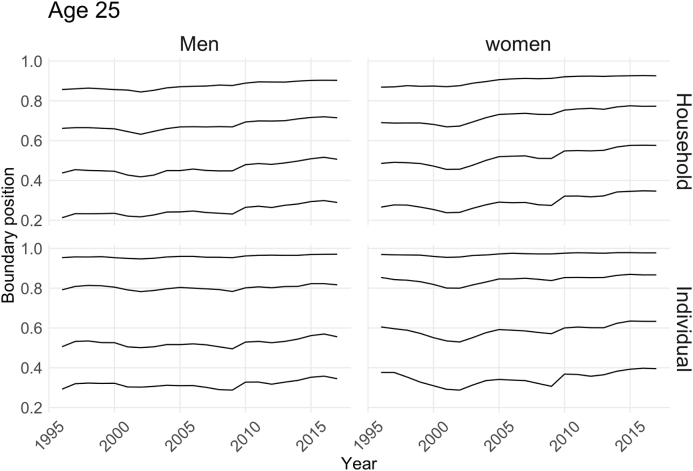
**Boundaries for age 25 between income quintiles using population cut-points.***Source:* Authors' calculation based on Finnish registry data, 1995–2017.

**Fig. A6:**
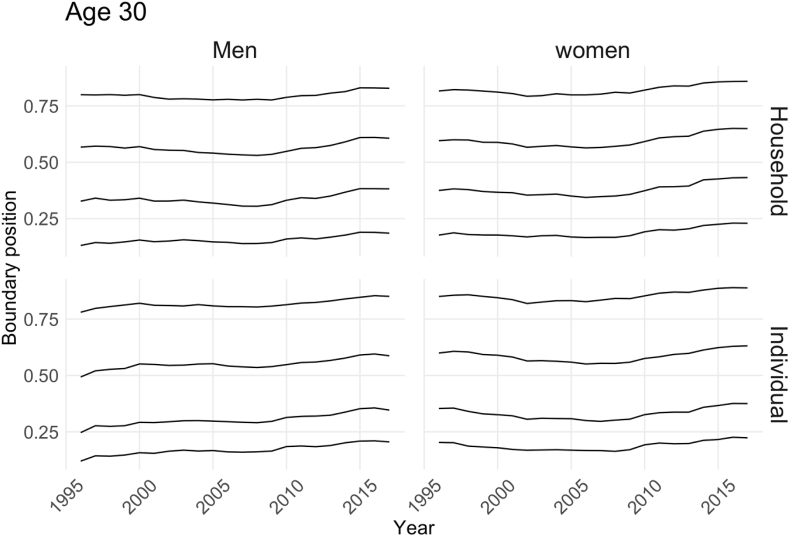
**Boundaries for age 30 between income quintiles using population cut-points.***Source:* Authors' calculation based on Finnish registry data, 1995–2017.

**Fig. A7:**
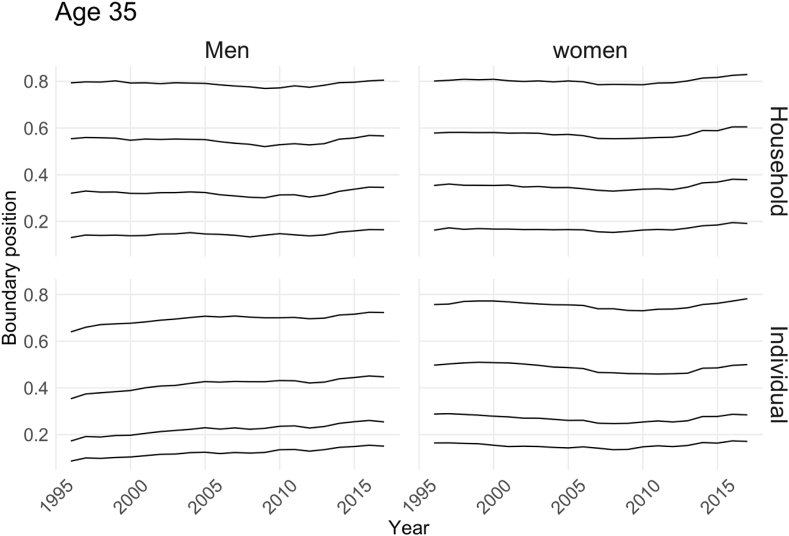
**Boundaries for age 35 between income quintiles using population cut-points.***Source:* Authors' calculation based on Finnish registry data, 1995–2017.

**Fig. A8:**
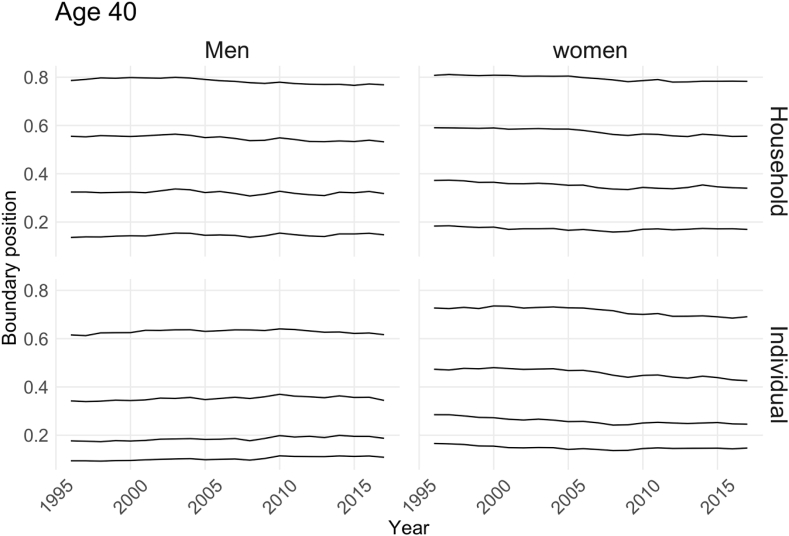
**Boundaries for age 40 between income quintiles using population cut-points.***Source:* Authors' calculation based on Finnish registry data, 1995–2017.

**Fig. A9:**
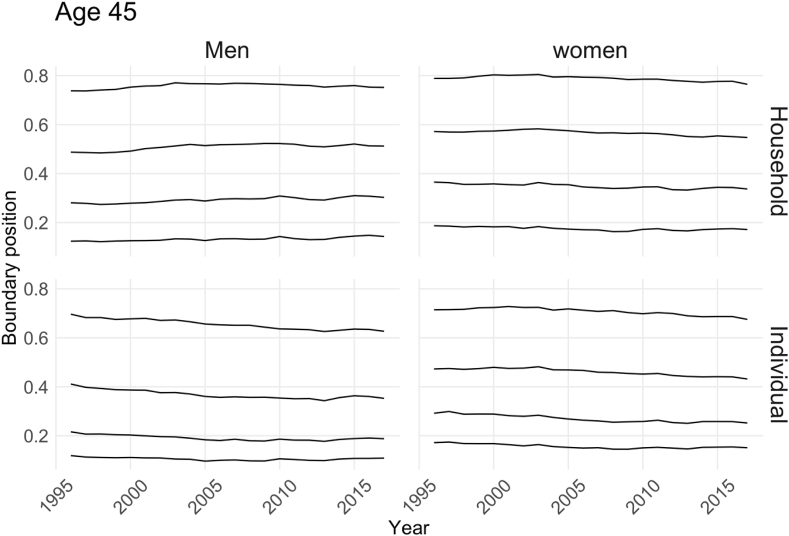
**Boundaries for age 45 between income quintiles using population cut-points.***Source:* Authors' calculation based on Finnish registry data, 1995–2017.

**Fig. A10:**
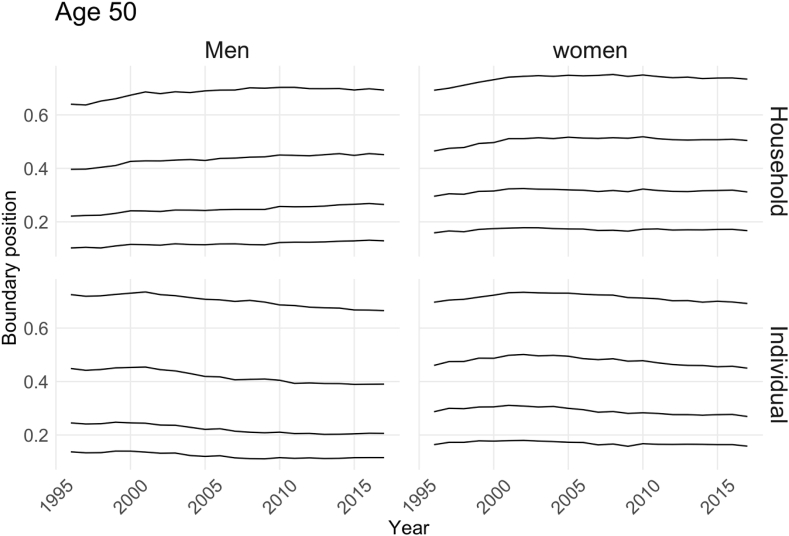
**Boundaries for age 50 between income quintiles using population cut-points.***Source:* Authors' calculation based on Finnish registry data, 1995–2017.

**Fig. A11:**
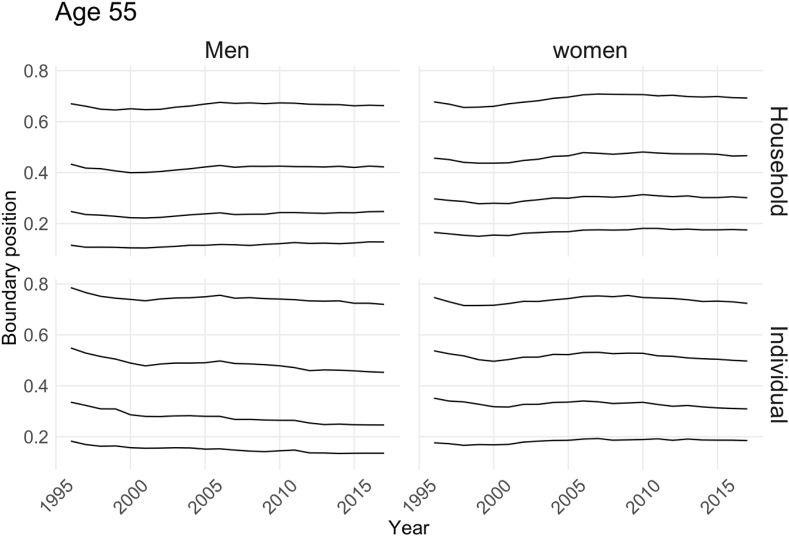
**Boundaries for age 55 between income quintiles using population cut-points.***Source:* Authors' calculation based on Finnish registry data, 1995–2017.

**Fig. A12:**
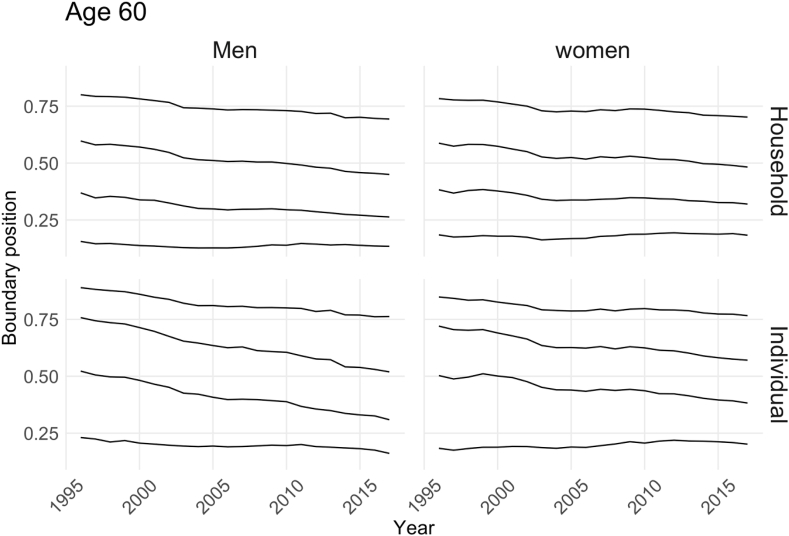
**Boundaries for age 60 between income quintiles using population cut-points.***Source:* Authors' calculation based on Finnish registry data, 1995–2017.

**Fig. A13:**
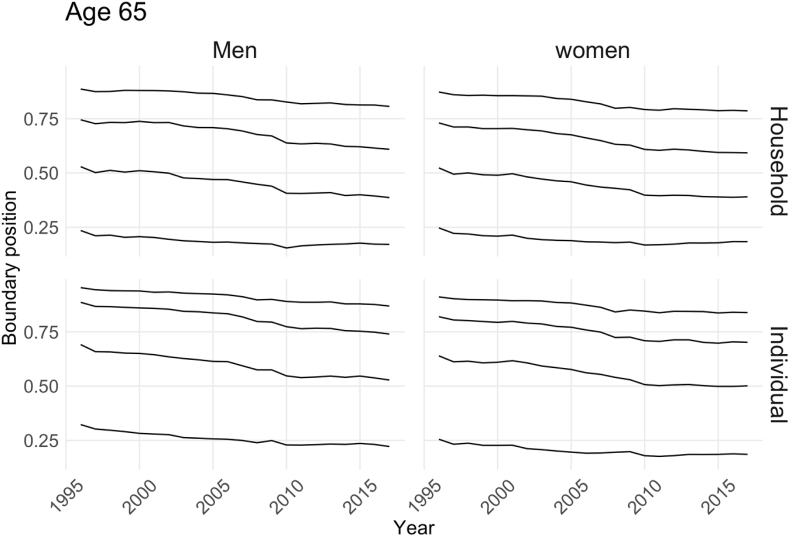
**Boundaries for age 65 between income quintiles using population cut-points.***Source:* Authors' calculation based on Finnish registry data, 1995–2017.

**Fig. A14:**
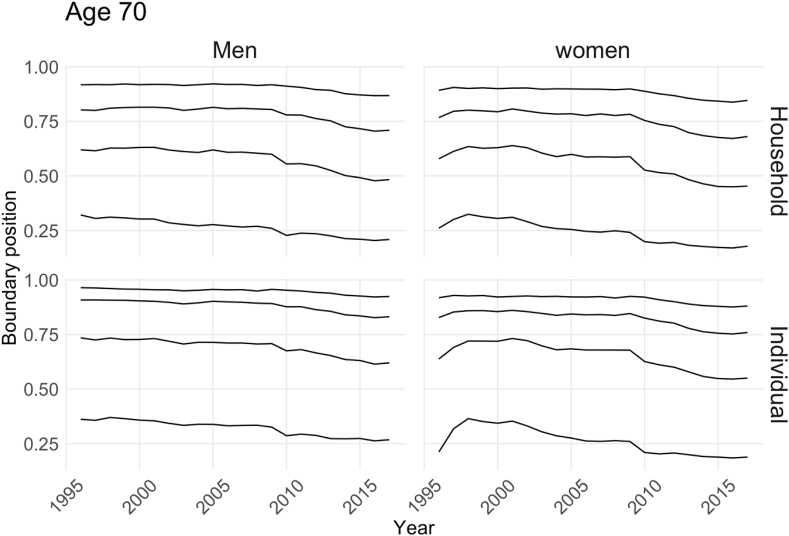
**Boundaries for age 70 between income quintiles using population cut-points.***Source:* Authors' calculation based on Finnish registry data, 1995–2017.

**Fig. A15:**
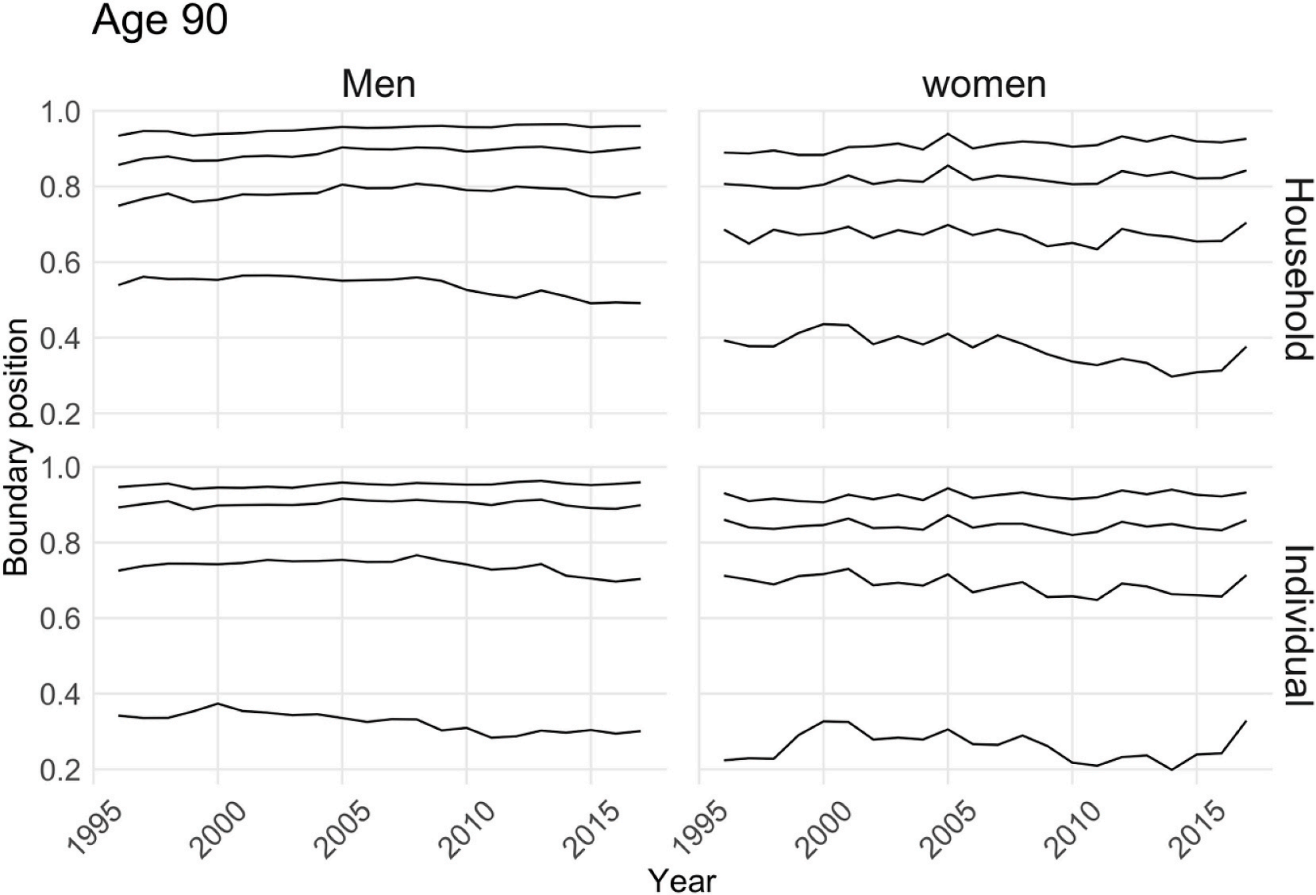
**Boundaries for age 75 between income quintiles using population cut-points.***Source:* Authors' calculation based on Finnish registry data, 1995–2017.

**Fig. A16:**
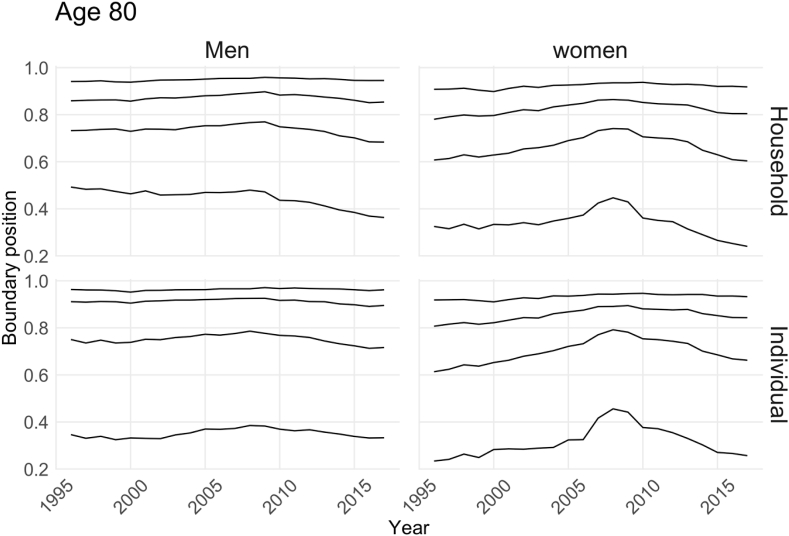
**Boundaries for age 80 between income quintiles using population cut-points.***Source:* Authors' calculation based on Finnish registry data, 1995–2017.

**Fig. A17:**
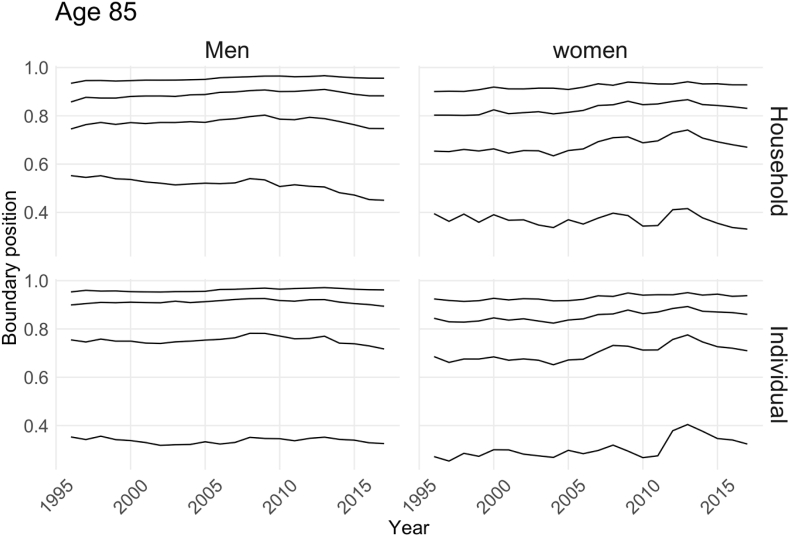
**Boundaries for age 85 between income quintiles using population cut-points.***Source:* Authors' calculation based on Finnish registry data, 1995–2017.

**Fig. A18:**
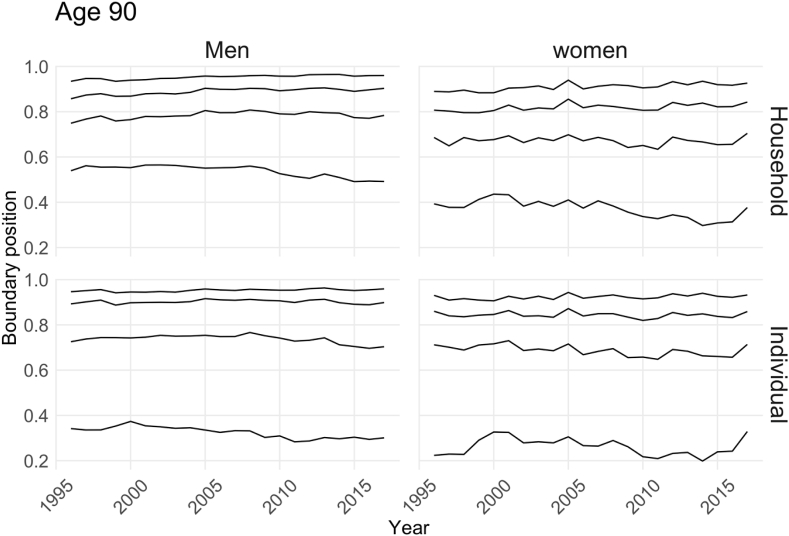
**Boundaries for age 90 between income quintiles using population cut-points.***Source:* Authors' calculation based on Finnish registry data, 1995–2017.

**Fig. A19:**
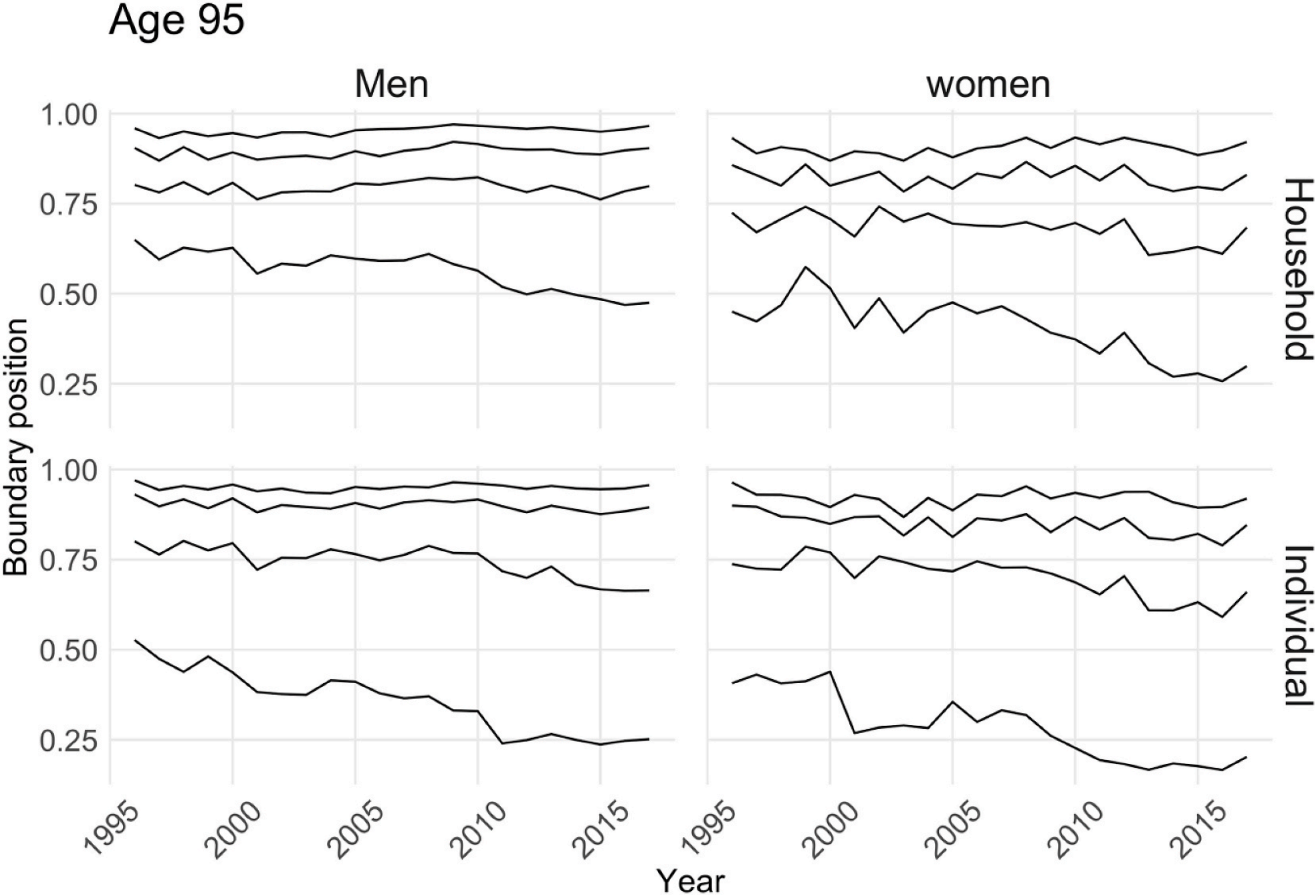
**Boundaries for age 95 between income quintiles using population cut-points.***Source:* Authors' calculation based on Finnish registry data, 1995–2017.

The increasing differences in mortality inequalities between age-specific and population-wide groupings might relate in part to the changing age patterns of the boundaries between income quintiles. A closer examination of these patterns showed that the boundary positions at ages below 60 were relatively stable across time (Fig. A5, Fig. A6, Fig. A7, Fig. A8, Fig. A9, Fig. A10, Fig. A11). This result suggests that it is unlikely that the temporal changes in mortality inequalities detected at ages below 60 when population groupings were used were caused by compositional changes related to the grouping method. On the other hand, larger temporal changes in the boundaries at ages above 60 were observed, particularly for women (Fig. A12, Fig. A13, Fig. A14, Fig. A15, Fig. A16, Fig. A17, Fig. A18, Fig. A19). The reduction in size of the lower quintiles at retirement ages in recent years is explained by increases in pension incomes among younger generations of retiring women (Rantala et al., 2017).

For women, the life expectancy ranking was consistently higher for the lowest quintile than it was for the second-lowest quintile when population-wide cut-points were used, which was not the case when age-specific cut-points were used. As the calculation of *SII* is based on the assumption that life expectancy increases monotonically by income quintile, the unexpected higher ranking of the lowest quintile's life expectancy with population-wide cut-points led to a lower *SII.* Thus, one might have expected the *SII* to be lower when the population-wide grouping is used than when the age-specific grouping is used. Nevertheless, because life expectancy was more dispersed overall across income quintiles using population-wide cut-points compared to age-specific cut-points, the *SII* in the population-wide operationalization was always larger than in the age-specific operationalization. Therefore, summary measures such as *SII* and *RII* should be carefully interpreted when the ranking of mortality outcomes is not consistently associated with the socio-economic ranking.

### 5.3 Limitations

**Fig. A20:**
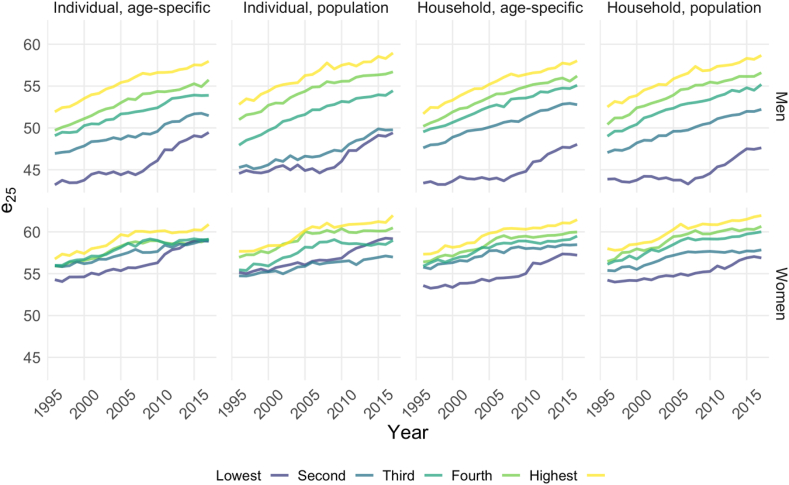
**Life expectancy by income quintile with gender-combined income ranking (row: gender, column: income definition).***Source:* Authors' calculation based on Finnish registry data, 1995–2017.

**Fig. A21:**
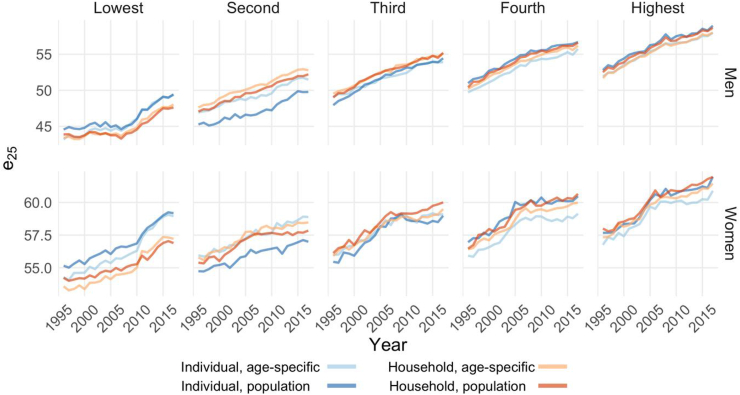
**Life expectancy by income definition with gender-combined income ranking (row: gender, column: income quintile).***Source:* Authors' calculation based on Finnish registry data, 1995–2017. Note: The scales for the first and the second row are different.

**Fig. A22:**
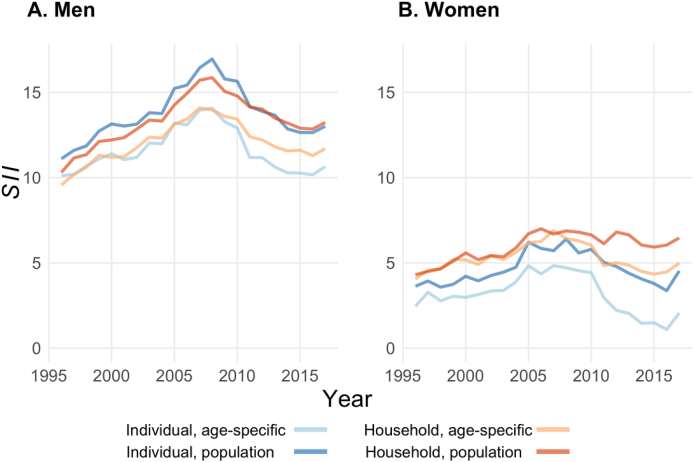
**Trends in the slope index of inequality for life expectancy by income measure and gender (gender-combined income ranking).***Source:* Authors' calculation based on Finnish registry data, 1995–2017.

**Fig. A23:**
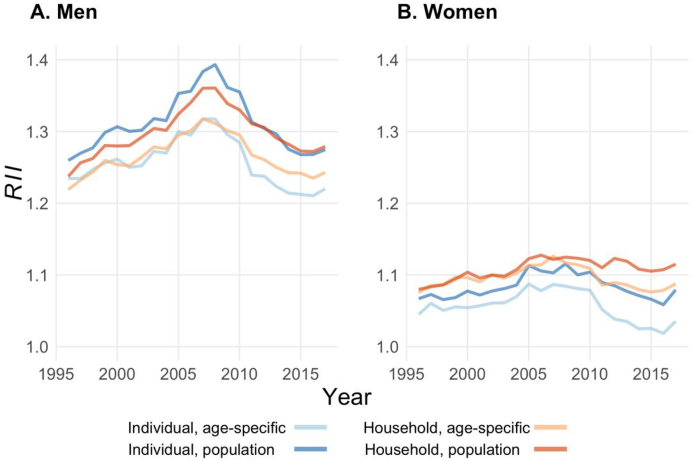
**Trends in the relative index of inequality for life expectancy by income definition and gender (gender-combined ranking).***Source:* Authors' calculation based on Finnish registry data, 1995–2017.

**Fig. A24:**
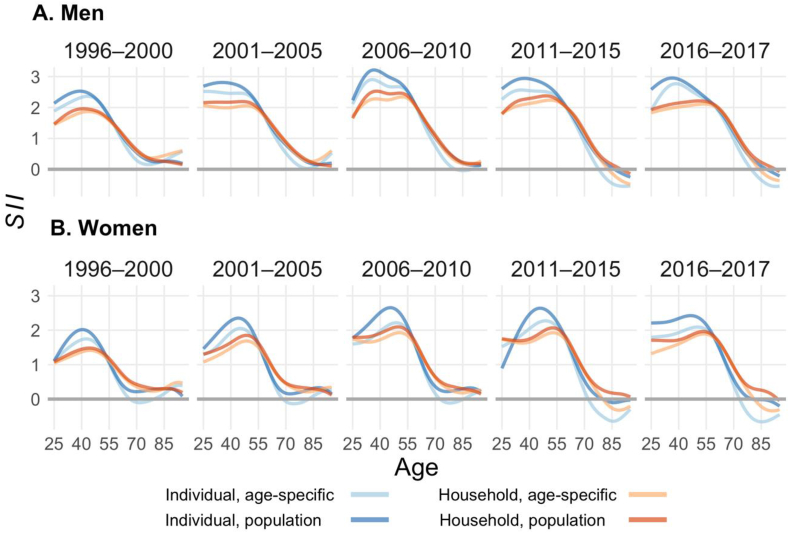
**Slope index of inequality for age-specific mortality rates by period and gender (gender-combined ranking).***Source:* Authors' calculation based on Finnish registry data, 1995–2017. To illustrate, a level of SII of 2 here means the difference in log mortality rate between people at the very top and the very bottom of the income distribution is 2.

**Fig. A25:**
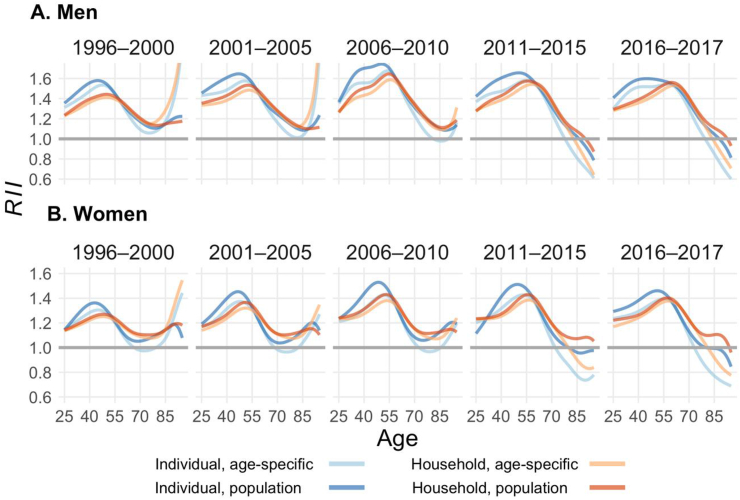
**Relative index of inequality for age-specific mortality rates by period and gender (gender-combined ranking).***Source:* Authors' calculation based on Finnish registry data, 1995–2017. *Note*: The scales for the upper row and the bottom row are different. To illustrate, a level of *RII* of 1.5 here means the ratio of log mortality rate between people at the very top and the very bottom of the income distribution is 1.5.

We are aware of several limitations to our study. First, we provided only four definitions of income, even though there are numerous additional ways of measuring income. For example, although the literature seems to mostly agree on the gender-specific income rankings, we might also be interested in exploring whether income positions should be measured with gender-combined income distributions, since the cost of most major living expenses does not differ by gender. We conducted these analyses as well and found that the gender-combined approach led to estimates (Fig. A20, Fig. A21, Fig. A22, Fig. A23, Fig. A24, Fig. A25) very close to the findings we have reported for gender-specific measures. Other options for measuring income include gross individual income and household income. However, as prior evidence shows that pre-tax income is more closely associated with mortality than post-tax measures, it is possible that studies that used pre-tax income definitions may have overstated the role of income for mortality differentials (Martikainen et al., 2009).

**Fig. A26:**
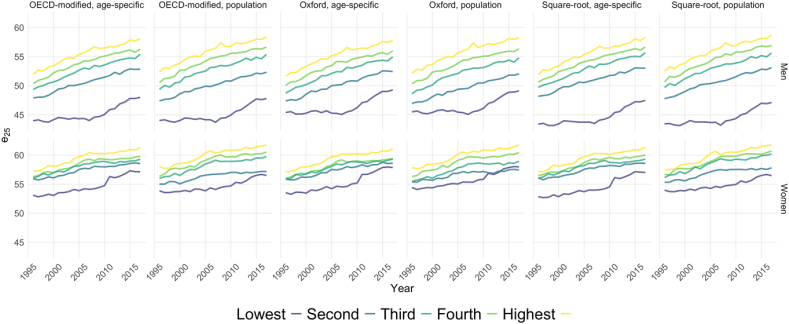
**Life expectancy trends by household disposable income using OECD-modified, Oxford, and square-root equivalence scales.***Note:* The OECD-modified scale assigns 1 to the first adult, 0.5 to each additional adult member, and 0.3 to each child (Hagenarrs et al., 1994). The “Oxford” scale assigns 1 to the first adult, 0.7 to each additional adult member, and 0.5 to each child (OECD 1982). The square-root scale divides household income by the square root of household size (OECD 2008, 2011).

**Fig. A27:**
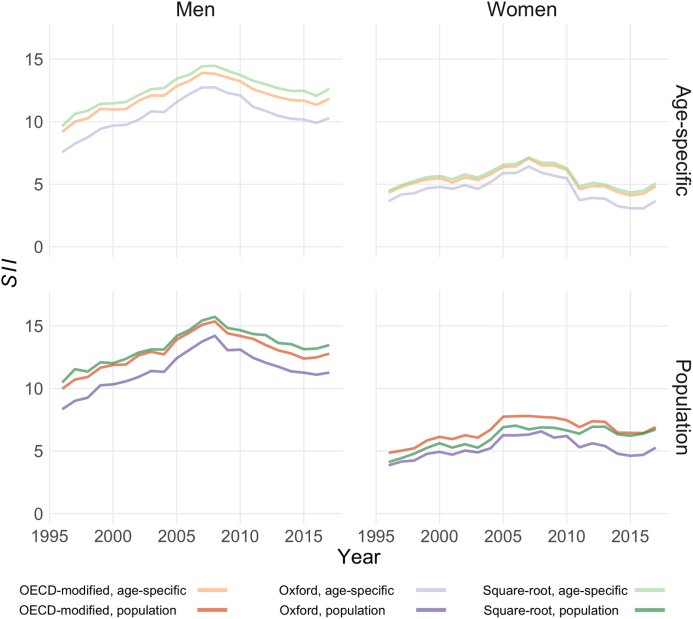
**Trends of slope index for life expectancy by household disposable income using OECD-modified, Oxford, and square-root equivalence scales.***Note:* The OECD-modified scale assigns 1 to the first adult, 0.5 to each additional adult member, and 0.3 to each child (Hagenarrs et al., 1994). The Oxford scale assigns 1 to the first adult, 0.7 to each additional adult member, and 0.5 to each child (OECD 1982). The square-root scale divides household income by the square root of household size (OECD 2008, 2011).

Further, although the OECD-modified scale has been widely used by researchers and organizations to calculate household disposable income in mortality inequality research (e.g., Brønnum-Hansen, Östergren, et al., 2021; OECD, 2013; Tarkiainen et al., 2012), alternatives exist. Previous studies have suggested that equivalence scales have an impact on the level of income inequality itself, as well as on the association between income and mortality (Dudel et al., 2021; Pascual et al., 2005). Additional analysis using the Oxford and square-root scales found largely the same temporal trends, although the magnitude of mortality inequalities varied substantially across the scales (Fig. A26, Fig. A27).

Additionally, it could be argued that the Finnish context (e.g., health and pension systems, income inequality) may limit the generalizability of our results. As Finland has relatively high life expectancy, our findings may be more relevant to other low-mortality countries. Prior comparative studies have found that the relationship between income and mortality or self-rated health in Finland is not unique, as similar patterns have been found in the US and other European countries (Elo et al., 2006; Mackenbach et al., 2005; Mortensen et al., 2016). In addition, the associations found in Finland between education and health outcomes, including mortality, morbidity, and self-rated health, are similar to the average levels observed in other northern and western European countries (Mackenbach et al. 2018, 2019). Hence, similar findings may be found in other low-mortality countries. Further research is necessary to examine how different income definitions affect mortality inequality estimates in other countries.

A full picture of the relationship between income and mortality cannot be achieved without understanding income inequality. Rising (falling) income inequality may increase (decrease) the mortality differences between individuals. If the income distribution had remained unchanged over time, we might conclude that it was the change in the “effect” of income that led to the trends in income quintile-mortality associations. In Finland, income inequality, as measured by the Gini coefficient of household disposable income, is low in international comparison. It rose steadily from 1995 to 2007, decreased slightly in the following two years, and remained relatively stable up to 2016 (the last year of income for the 2017 life tables; OECD, 2021). This pattern suggests (1) that the increase in the association between income quintiles and life expectancy over the 1996–2008 period may be partly explained by the rise in income inequality; and (2) that the decline in the association between income quintiles and life expectancy from 2008 onwards has been more related to mechanisms other than income inequality, such as the declining importance of income.

We assumed that low income causes higher mortality, and interpreted our results mostly following this rationale. However, direct (reverse causality) and indirect selection (due to early life adversity, education, etc.) also contribute to mortality inequalities (Blane et al., 1993; West, 1991). Reverse causality might partially explain why mortality inequalities were smaller at ages over 60, given that poor health does not impact retirement income to the extent that it impacts labor market income and mortality in working age. The net effect of income on health and the net effect of health on income may be disentangled, for example, by performing individual-level analyses of change and/or utilising natural experiment designs. We used the income from the previous year, but future research can use longer lags of income to test relevant hypotheses.

### 5.4 Lessons for future research

What insights can researchers gain from our results? Researchers should bear in mind that any findings reported on the association between income and health in the literature are strongly affected by how income is conceptualized and operationalized. It is often the case that researchers cannot test and use all of the alternative definitions because of data limitations. Our results may thus help researchers make reasonable predictions of what the results would be if alternative definitions were used.

It is reassuring that, for both genders, the age patterns were approximately the same regardless of which income definition was applied. Yet the use of different income definitions can have different implications across ages. For instance, the results for household and individual income definitions tended to converge at older ages, possibly because of the convergence of income differences or the emergence of biological frailty associated with old age. The choice of income definitions appears to have more implications for women than for men, as we found inconsistent period trends and more variations in mortality inequality levels across income definitions for women than for men.

Finally, this work can be extended in at least two ways. First, income may be included in a socio-economic summary indicator, such as the affluence index (Cairns et al., 2019) to analyse mortality. Second, there has been growing interest in inequalities in other mortality outcomes, such as the modal age at death, lifespan inequality, and the distance between lifespan distributions (Brønnum-Hansen, 2017; Shi et al., 2021; van Raalte 2018), as well as in morbidity outcomes such as healthy life expectancy (Jagger et al., 2020). Future research is needed to examine how the use of different income definitions may affect these two lines of measurement and research.

## 6 Conclusions

We showed that applying the four income definitions often used in health inequality research led to substantial differences in the estimated levels of income-specific life expectancy and the magnitudes of inequality in life expectancy. These results indicate that caution is needed when interpreting the findings of analyses on the association of income with mortality, including efforts in science communication. It is likely that the choice of income definition may lead to an increase or decrease of the reported life expectancy differences by several years. The reassuring news is that the age patterns observed in this study were robust for both genders. However, while the period trends were shown to be stable for men, significant differences were observed for women. Researchers should base their decisions about which income definition to use in their analyses on conceptual and theoretical considerations.

## Ethical statement

The study has been approved by the Statistics Finland Board of Statistical Ethics (TK-53-1121-18).

## Footnotes

1 When computing household disposable income, different scales are available such as the Oxford scale and the OECD-modified scale, which use different weights for additional family members.

2 Note that the patterns of the *SII* and the *RII* for life expectancy shown here should not be assumed to be the same as the *SII* and the *RII* for rate measures, such as the age-standardized mortality rate that has been used by prior research (e.g., Mackenbach et al., 2018).
